# How informative is your kinetic model?: using resampling methods for model invalidation

**DOI:** 10.1186/1752-0509-8-61

**Published:** 2014-05-22

**Authors:** Dicle Hasdemir, Huub CJ Hoefsloot, Johan A Westerhuis, Age K Smilde

**Affiliations:** 1 Biosystems Data Analysis Group, Swammerdam Institute for Life Sciences, University of Amsterdam, Amsterdam, The Netherlands; 2 2Netherlands Metabolomics Centre, Leiden, The Netherlands

**Keywords:** Model invalidation, Kinetic models, ODE, Differential equations, Smooth principal components analysis, SPCA, PCA, Resampling, Cross validation, Forecast analysis

## Abstract

**Background:**

Kinetic models can present mechanistic descriptions of molecular processes within a cell. They can be used to predict the dynamics of metabolite production, signal transduction or transcription of genes. Although there has been tremendous effort in constructing kinetic models for different biological systems, not much effort has been put into their validation. In this study, we introduce the concept of resampling methods for the analysis of kinetic models and present a statistical model invalidation approach.

**Results:**

We based our invalidation approach on the evaluation of a kinetic model’s predictive power through cross validation and forecast analysis. As a reference point for this evaluation, we used the predictive power of an unsupervised data analysis method which does not make use of any biochemical knowledge, namely Smooth Principal Components Analysis (SPCA) on the same test sets. Through a simulations study, we showed that too simple mechanistic descriptions can be invalidated by using our SPCA-based comparative approach until high amount of noise exists in the experimental data. We also applied our approach on an eicosanoid production model developed for human and concluded that the model could not be invalidated using the available data despite its simplicity in the formulation of the reaction kinetics. Furthermore, we analysed the high osmolarity glycerol (HOG) pathway in yeast to question the validity of an existing model as another realistic demonstration of our method.

**Conclusions:**

With this study, we have successfully presented the potential of two resampling methods, cross validation and forecast analysis in the analysis of kinetic models’ validity. Our approach is easy to grasp and to implement, applicable to any ordinary differential equation (ODE) type biological model and does not suffer from any computational difficulties which seems to be a common problem for approaches that have been proposed for similar purposes. Matlab files needed for invalidation using SPCA cross validation and our toy model in SBML format are provided at http://www.bdagroup.nl/content/Downloads/software/software.php.

## Background

With the concept of ‘sytems biology’ coming to the stage of biological research, construction of kinetic models has been the primary focus in a substantial number of studies [1-4]. Kinetic models are mechanistic representations of biological systems. They include information on two main levels. The first level of information includes the metabolites, enzymes, signaling molecules and chemical reactions involved in the model together with the formulation of the reaction kinetics such as Michaelis-Menten kinetics. Knowledge about inhibition, activation and allosteric regulation of enzymes are also a part of this level. The second level of information consists of numerical values of all different parameters defined in the first level of information. Those parameters include but are not limited to rate parameters for chemical reactions such as production of new metabolites in metabolic models, post-translational modifications of proteins in signaling pathways and transcription processes in genetic regulatory circuits.

As of present, kinetic models are usually restricted to small scale systems. The median of the number of the reactions and species that 462 curated kinetic models in Biomodels Database [5] included are only 12 and 11, respectively. Yet the information they provide at both levels increases very rapidly. This is usually accomplished by *in vitro* experiments which give insight into appropriate formulations of enzyme kinetics. Also values of the parameters can be determined by *in vitro* experiments with isolated enzymes. Another common way towards this aim is the use of *in vivo* experiments in which metabolite concentrations are measured. Optimal values of the parameters can then be estimated by using concentration data [6]. However, *in vitro* and *in vivo* kinetics can be very different, not only in the values of the parameters but more importantly, also in the formulation [3]. This points to the need for careful investigation of the model’s validity on the first information level that we defined above.

Most of the time, models are assessed qualitatively based on the goodness of their fit to concentration data [2]. In some other cases, new datasets in different biological conditions are generated and a qualitative analysis is made based on the model’s ability to predict new datasets [7]. However, most of the time multiple candidate models with different structures can show very similar goodness of fit and also prediction in another experimental condition. This stems from high levels of adaptability in these models. One could argue that all candidate models are good as long as they perform reasonably well in prediction. However, rapid elimination of less informative models would be very beneficial to the metabolic modeling community. It would ease the way to trustworthy libraries of models providing the researchers with speed and accuracy for larger scale models. To this aim, model selection and invalidation algorithms supply a quantitative framework.

Model selection criteria borrowed from statistical literature such as Akaike and Bayesian Information Criteria (AIC and BIC respectively) are among the most popular approaches introduced for the selection of sytems biology models [8-10]. Model selection based on AIC have also been successfully implemented in software packages which aim to select the best model within a family of automatically generated models derived from one master model by adding/removing species or interactions [11,12].

However, those criteria always support in favor of one model without providing any significance to their decisions [13] and can not produce clear results when many parameters are involved [12]. An alternative which is capable of ranking different models according to their plausibility was introduced within a Bayesian perspective using Bayes Factors [14]. This family of Bayesian methods unfortunately still remain unemployed in the field due to the need for smart assumptions on parameters’ prior distributions and their costliness in calculation of bulky integrals despite promising effort regarding the second obstacle [15,16]. In some studies robustness based measures were proposed for model selection [17,18]. For oscillating systems, robustness of the model can support its preference over different models. However, this might not hold true for the whole family of kinetic models in systems biology.

Although not employed regularly, the systems biology community has been provided with tools to select between different model structures. However, invalidation of a model structure without an alternative to compare with has not been considered much in the related literature. An analytical approach suggests use of barrier certificates which are functions whose existence proves that the model behaviour can never intersect the experimental data [19]. The existence of the barrier certificates proves the invalidity of the models. However the approach is purely analytical and very complex so it is not easily applicable by biologists. Another drawback is the difficulty in the construction of the barrier certificates for complicated system descriptions as the authors also elaborate in their paper.

In this article, we introduce a statistical measure for the invalidation of kinetic models which suffers neither from complex model descriptions nor large scale models. We use the predictive power of Smooth Principal Components Analysis (SPCA), an unsupervised data analysis method as a threshold to assess the predictive power of kinetic metabolic models. By using this threshold value, we can determine which model structures are informative enough to deserve further attention and which model structures should be abandoned. Our approach stands on a basic assumption: If a totally unsupervised data analysis method without any prior biochemical knowledge predicts better than a kinetic model can do, that points to an inaccuracy or incompleteness in the information which the kinetic model provides us with.

With this paper, we also want to bring the attention of the systems biology community to the idea of using resampling methods which have proven to be very useful in machine learning and data analysis. To our knowledge these methods’ potential has not been exploited fully in the analysis of kinetic systems biology models.

Using synthetic data generated from metabolic models has been adopted widely in literature as a way of testing algorithms in a controlled context [20]. Here, we also employed this approach and used a toy metabolic model and a real signaling model for the generation of data. By using this data, we tested models also with lower complexity than the true model to assess the sensitivity and specificity of our approach.

We applied our method also on an eicosanoid production model in human white blood cells. Eicosanoid is a subclass of fatty acyls. Fatty acyls constitute one of the six major classes of lipids and are related to inflammation, rheumatoid arthritis, sepsis and asthma. Eicosanoids are divided into different groups one of which is prostaglandin family. Prostaglandins have been found to be related to many symptoms of inflammation like fever and pain [2,21,22]. That makes the eicosanoids important targets for modeling studies which can be used for predictive purposes in response to treatment with anti-inflammatory drugs. A kinetic model describing the production of prostaglandins from Arachidonic acid has been published in [2]. The model includes the substrate Arachidonic Acid, 8 downstream metabolites, signaling molecules and 4 different enzymes. All reactions were formulated by mass action kinetics. Due to the scarcity of information on enzyme activity regulation, rate parameters for enzymatic reactions were formulated as linear functions of enzyme-regulator molecules. Given the simplicity of the kinetics in the model and limited number of components, we wanted to assess its informative level and our results showed that the model could not be invalidated with the available data.

The other benchmark pathway we analysed was the well known high osmolarity glycerol (HOG) pathway in yeast. Osmo-adaptation in yeast has started to receive increasing attention with the discovery of the associated mitogen-activated protein kinase (MAPK) cascade [23,24]. Since then, the HOG pathway proved to be a well studied model system to study the principles of signal transduction due to MAPK cascades being conserved eukaryotic signal transduction pathways. The pathway is in charge of regulating the glycerol accumulation in the cell in response to the changing osmotic pressure in the environment. It has been widely accepted that the upstream pathway consists of two redundant paths starting with two different transmembrane osmosensor proteins Sho1p and Sln1p. The cascade proceeds with the phosphorylation of Pbs2p, Pbs2p-Sho1p complex and Hog1p towards the transcriptional regulation of glycerol production [25,26]. However, there is still active debate on post-translational interactions and transient feedback mechanisms involved in the signal transduction [26,27]. Therefore we analysed a recently published comprehensive model of the HOG pathway to check its predictive properties given part of the experimental data used to build the model [26,27]. We also used the model as a basis for our simulation studies in which we generated data according to the published level of complexity and questioned a simplified version for its validity.

## Methods

### Toy metabolic model

**Figure 1:**
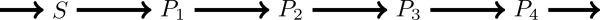
**Layout of the toy model. **Unbranched toy model consisted of one substrate and 4 downstream products.

We used an unbranched toy metabolic pathway for the generation of synthetic data (Figure 1). It included one substrate and four downstream metabolites whose production followed Michaelis-Menten kinetics. Equation 1 shows the set of ordinary differential equations constituting the true model (*ODE*_*T*_) which we used for the generation of the data. We used the dynamic part of the time series data in the first 22 time points as the data without experimental noise. We stored the data in a matrix with metabolites in the columns and time points in the rows.

We introduced homogeneous experimental noise to the data in the form of Gaussian noise with zero mean and varying standard deviation. We varied the standard deviation of noise between 0.001 and 0.05. At each degree of experimental noise, we repeated the simulations with 100 different realizations of the data.

(1) $$\begin{array}{lll} \frac{\text{dS}}{\text{dt}} & =k_{\text{in}}-\frac{v_{{\text{max}}_{1}}\left[S\right]}{{\text{Km}}_{1}+\left[S\right]}\; & \\ \frac{\text{dP}1}{\text{dt}} & =\frac{v_{{\text{max}}_{1}}\left[S\right]}{{\text{Km}}_{1}+\left[S\right]}-\frac{v_{{\text{max}}_{2}}\left[P1\right]}{{\text{Km}}_{2}+\left[P1\right]}\; & \\ \frac{\text{dP}2}{\text{dt}} & =\frac{v_{{\text{max}}_{2}}\left[P1\right]}{{\text{Km}}_{2}+\left[P1\right]}-\frac{v_{{\text{max}}_{3}}\left[P2\right]}{{\text{Km}}_{3}+\left[P2\right]}\; & \\ \frac{\text{dP}3}{\text{dt}} & =\frac{v_{{\text{max}}_{3}}\left[P2\right]}{{\text{Km}}_{3}+\left[P2\right]}-\frac{v_{{\text{max}}_{4}}\left[P3\right]}{{\text{Km}}_{4}+\left[P3\right]}\; & \\ \frac{\text{dP}4}{\text{dt}} & =\frac{v_{{\text{max}}_{4}}\left[P3\right]}{{\text{Km}}_{4}+\left[P3\right]}-k_{\text{out}}\left[P4\right]\; \end{array}$$

### Comparison of predictive power by cross validation

**Figure 2:**
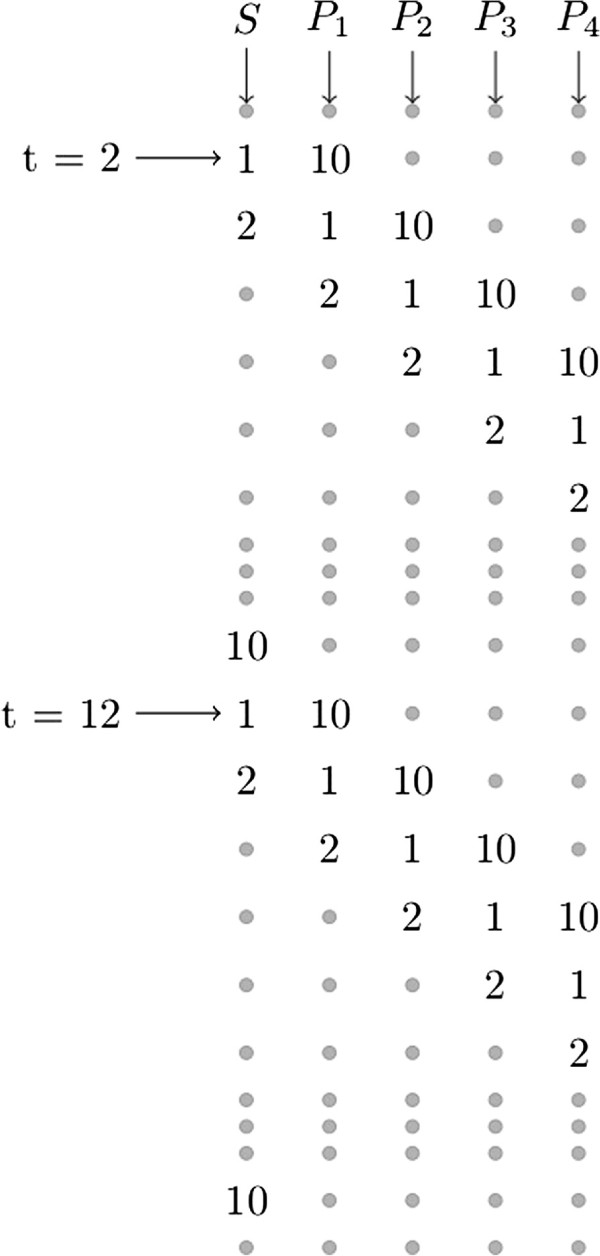
**Stratified diagonal cross validation scheme. **1 denotes the elements in the first test set whereas 2 denotes those in the second test set and so on. Elements of 10 different test sets were diagonally selected as shown in the figure.

One of the key features of our approach is using cross validation, a resampling technique as we mentioned in our introduction. Cross validation is a very commonly used validation method in statistics and machine learning [28,29] for determining the optimal level of complexity in models. In cross validation, a data set is divided into two parts: training and test sets. Only the training set is used for the parameter inference whereas the test set is only used for assessing the performance of the model on parts of the data that have not been associated with parameter inference. The procedure is repeated with alternating training and test sets several times and the performance results are averaged over all repetitions. In classification problems, that performance measure is the accuracy in classification of the test objects. In regression or dimension reduction problems, it is the prediction error. Throughout this paper we refer to the residuals in the prediction of only the test set data points by using the term ‘prediction error’. In this study, we inferred the parameters of both the kinetic and the SPCA model using the training data and we used prediction error as a measure of the predictive power of both modeling approaches.We used a diagonal cross validation scheme in which 10% of the data was used as the test set. This kind of stratified cross validation scheme provided us with diverse test sets which were homogeneous both in metabolites and time points (Figure 2). With this scheme, every element- excluding the first and the last time points- in the data matrix belonged to a test set once and the sum of the prediction error over all test sets gave the total prediction error. The first time points were not included in the test sets because initial concentrations of the metabolites were also unknown parameters of the kinetic model as we will touch upon also in the proceeding sections. That is why these points could not be used as test points in cross validation. The reason for excluding the last time points was related to the fact that it is more challenging to predict the end points with SPCA compared to the interior time points. Due to this fact, we approached the prediction of last time points in the forecast analysis context where we could adjust the smoothing penalty parameter of SPCA accordingly.

### Comparison of predictive power by forecast analysis

Forecasting refers to predicting the future outcome of a variable of interest. It is commonly used in a lot of disciplines ranging from economics to meteorology where modeling is crucial. In forecast analysis, models are established using past data and extrapolated to the future. Variations on forecast analysis exist depending on the types of the models, the needs of the field, partitioning of the training and test sets and the types of the measures that are used to assess the amount of prediction error [30].

Here, we used a basic scheme which fits for both SPCA and kinetic modeling. In each run, we left out approximately the last 20% of the time points of one metabolite as the test set. By this way, we could assign a certain percentage of the end time profiles to a test set once and the total prediction error on those time points gave us a measure for the predictive power of the models.

### Kinetic modeling

We estimated the rate parameters $\left(\vec{k}\right)$ and the initial metabolite concentrations at $t=0\;({\vec{X}}_{0})$ from the training data. We carried out the optimization with a nonlinear solver in Matlab, namely lsqnonlin function which implements the trust-region-reflective algorithm [31]. The objective function was to minimize the square of the difference between the noisy synthetic data and the model values of the training set elements. In Equation 2, the weighting matrix ***W***_***tr ***_is a binary matrix with 0’s corresponding to the test set elements in the data matrix and 1’s corresponding to the training set elements. We excluded test set elements from the parameter inference process by element-wise multiplication by ***W***_***tr***_. This multiplication is denoted by the Hadamard Product, ∘, whereas the model concentration values $\left(\hat{X}\right)$ are given as a function of the unknown parameter vectors $\vec{k}$ and ${\vec{X}}_{0}$.

(2) $$\begin{array}{l} \underset{\vec{k},{\vec{X}}_{0}}{\text{min}}{∥W_{\text{tr}}\circ \left(X-\hat{X}\left(\vec{k},{\vec{X}}_{0}\right)\right)∥}^{2} \end{array}$$

We estimated the model concentration values by numerically integrating the set of differential equations defining the model in question at every iteration step throughout the optimization. We repeated the procedure with two different models: the true model (Equation 1) and the simplified model (Equation 4). The true model (*ODE*_*T*_) is the model we had used for data generation. In the simplified model (*ODE*_*S*_), the production of the first metabolite was formulated with linear kinetics with only one rate parameter.

(3) $$\begin{array}{l} {∥W_{\text{test}}\circ \left(X-\hat{X}\left(\vec{k},{\vec{X}}_{0}\right)\right)∥}^{2} \end{array}$$

The prediction error for one test set was then calculated as in Equation 3 where ***W***_***test ***_has 0’s for training set elements and 1’s for test set elements.

(4) $$\begin{array}{lll} \frac{\text{dS}}{\text{dt}} & =k_{\text{in}}-k\left[S\right]\; & \\ \frac{\text{dP}1}{\text{dt}} & =k\left[S\right]-\frac{v_{{\text{max}}_{2}}\left[P1\right]}{{\text{Km}}_{2}+\left[P1\right]}\; & \\ \frac{\text{dP}2}{\text{dt}} & =\frac{v_{{\text{max}}_{2}}\left[P1\right]}{{\text{Km}}_{2}+\left[P1\right]}-\frac{v_{{\text{max}}_{3}}\left[P2\right]}{{\text{Km}}_{3}+\left[P2\right]}\; & \\ \frac{\text{dP}3}{\text{dt}} & =\frac{v_{{\text{max}}_{3}}\left[P2\right]}{{\text{Km}}_{3}+\left[P2\right]}-\frac{v_{{\text{max}}_{4}}\left[P3\right]}{{\text{Km}}_{4}+\left[P3\right]}\; & \\ \frac{\text{dP}4}{\text{dt}} & =\frac{v_{{\text{max}}_{4}}\left[P3\right]}{{\text{Km}}_{4}+\left[P3\right]}-k_{\text{out}}\left[P4\right]\; \end{array}$$

### Smooth principal components analysis

The other key feature of our approach is its comparative nature. The reference method we used for comparison was Smooth Principal Components Analysis (SPCA) [32]. SPCA is an extension of the well known dimension reduction method Principal Components Analysis (PCA) [29,33] with roughness penalties on the scores.

The reference method is completely unsupervised, making no use of the kinetic model structure nor of any prior biochemical knowledge. Smooth Principal Components Analysis penalizes the non-smoothness of the scores and thus can make use of the underlying time profile in predicting the missing points in the data [32]. This makes it more efficient than normal PCA to be used as a prediction method when the scores are expected to have smoothness as in the case of time series data.

We have estimated the smooth scores (**Z**) and loadings (**P**) within a Weighted Principal Components Analysis (WPCA) formulation. WPCA is a special variety of PCA in which data points are weighted proportional to the measurement accuracy at those points by using a weighting matrix [34]. WPCA can also be used to handle PCA on data with missing points using a binary weighting matrix where the entries corresponding to missing points are 0 [35]. That allows it to be employed as a favorite analysis method in multivariate statistics when there are missing points in the data [36] and also for performing cross validation where some of the data points are excluded as test set elements [28]. Our application in this study follows the latter.

We have minimized the objective function in Equation 5 by using the same nonlinear solver as we have used for kinetic modeling. The objective function in Equation 5 is comprised of two terms. The first term is the sum of squares of the difference between the measured (**X**) and model values of the training set elements by the SPCA model (**ZP**^*T*^). Here, ***W***_***tr ***_is the same binary matrix as we used in the Kinetic modeling Section. The second term is the penalty term scaled with the smoothing parameter *λ* where ***D***_***2***_ represents a second order difference matrix. With a second order penalty, scores are penalized for the change in slope [32] which is appropriate in our case since we deal with time series data.

(5) $$\underset{\text{Z},\text{P}}{\text{min}}{∥W_{\text{tr}}\circ \left(X-ZP^{T}\right)∥}^{2}+\lambda {∥D_{2}Z∥}^{2}$$

Prior to using SPCA, the number of principal components (PCs) and the value of the smoothing parameter (*λ*) have to be calibrated for each specific problem. We used cross validation also for this purpose. After the test set elements (outer test sets) which we used also in the Kinetic modeling Section were removed from the dataset, the remaining part was again subjected to a division of test (inner test sets) and training sets for a 10-fold cross validation with 10 repetitions. We applied SPCA using a particular value for *λ* and a particular number of PCs on every training set. The average prediction error on all different inner test sets from 10 different repetitions gave us a measure of how well the inner test set points could be predicted using that particular parameter combination. We repeated the same procedure by using increasing *λ* values and increasing number of PCs until the predictions on the inner test sets could not improve with increasing number of PCs and started to deteriorate with increasing *λ* after certain limits. These limits gave us the optimal values for the parameters. This approach is known as “Double Cross Validation” since it makes use of cross validation at two different levels and it leads to unbiased prediction errors [37].

**Figure 3:**
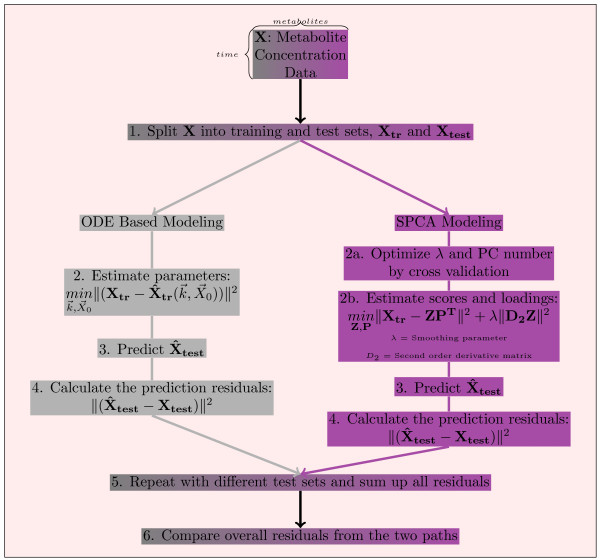
**Flowchart for the approach. **The figure summarizes graphically our comparative model invalidation approach.

Once the optimal *λ* and the optimal number of PCs were determined, they were used for the estimation of the scores (**Z**) and the loadings (**P**). Equation 6 shows how we calculated the prediction error for a single test set whether an inner or an outer test set. In Equation 6, ***W***_***test ***_has 1’s for test set elements and 0’s for training set elements as we used in the Kinetic modeling Section. In Figure 3, we give a detailed flowchart of our approach. 

(6) $${∥W_{\text{test}}\circ \left(X-ZP^{T}\right)∥}^{2}$$

In forecast analysis we followed the same approach with a small variation. There, we left out windows of data which consisted of 5 consecutive time points from the same metabolite as inner test sets, in each run. This helped us to infer the optimal parameters better for the accurate prediction of the end time points. This was because, also in forecast analysis, the purpose was to predict consecutive time points, in opposition to cross validation where the outer test set points were not consecutive.

## Results and discussion

### Toy model

We carried out simulations at different experimental noise levels. At the lowest noise level we tested, the experimental noise was drawn from a normal distribution with a standard deviation (*σ*_*noise*_) of 0.001. At this level of standard deviation, the mean relative noise in all of the 100 different realizations of the data was below 1%. At the maximum noise level (*σ*_*noise *_= 0.05), the mean relative noise at a single realization of the data could increase up to 13%. Mean Relative Noise (MRN) is a measure of the noise in the data calculated as the mean of individual relative noise levels for each element in the data matrix (Equation 7). In Equation 7, $x_{\text{ij}}^{m}$ denotes the values generated by the model according to Equation 1 whereas *x*_*ij *_denotes the synthetic data with experimental noise added.

(7) $$\begin{array}{lll} \text{MRN} & =\frac{\overset{n}{\underset{i=1}{\sum }}\overset{m}{\underset{j=1}{\sum }}\frac{\left|x_{\text{ij}}^{m}-x_{\text{ij}}\right|}{x_{\text{ij}}^{m}}}{n\times m}\; & \\ & \;n=\#\;\text{time points}\; & \\ & \;m=\#\;\text{metabolites}\; \end{array}$$

Tables 1 and 2 show all the invalidation decisions made in the simulations study. Results show that our SPCA-based comparative approach performs very well in invalidating simplified models, indicating the method’s high sensitivity. The low number of invalidation decisions made for the true model relate to the high specificity of our approach.

**Table 1:** All the invalidation decisions made by using cross validation

***σ***_***noise***_	**MRN (%)**	***ODE***_***S***_	***ODE***_***T***_
0.001	<1	100	0
0.01	2.2	100	0
0.025	5.4	100	4
0.03	6.5	100	8
0.05	10.8	75	14

The numbers show in how many of the 100 different noise realizations an invalidation decision was made for the models being questioned by using cross validation. *ODE*_*S *_and *ODE*_*T *_denote the simplified model and the true model, respectively. Mean Relative Noise at each noise level is given as the mean of the MRN values in 100 different realizations of the data, calculated based on Equation 7.

**Table 2:** All the invalidation decisions made by using forecast analysis

***σ***_***noise***_	**MRN (%)**	***ODE***_***S***_	***ODE***_***T***_
0.001	<1	100	0
0.01	2.2	100	3
0.025	5.4	86	17
0.03	6.5	81	17

The numbers show in how many of the 100 different noise realizations an invalidation decision was made for the models being questioned by using forecast analysis. *ODE*_*S *_and *ODE*_*T *_denote the simplified model and the true model, respectively. Mean Relative Noise at each noise level is given as the mean of the MRN values in 100 different realizations of the data, calculated based on Equation 7.

**Figure 4:**
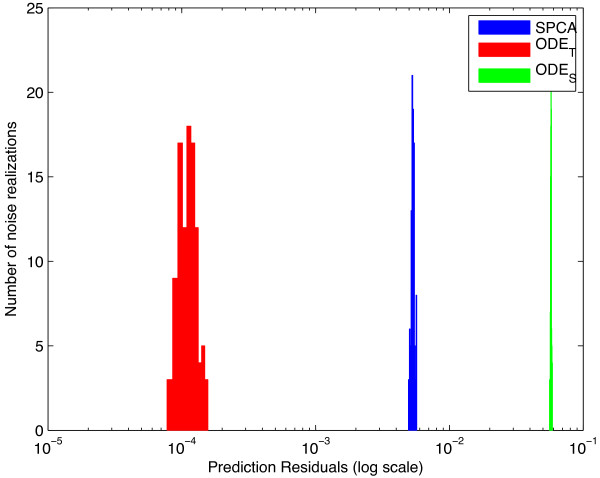
**Prediction errors in the simulations with very low level of noise. **The figure shows the residuals obtained in the cross validation simulations at the lowest noise level. At very low noise levels, there is a clear difference between prediction errors of the true and the simplified models both in cross validation and forecast analysis. The figure has a logarithmic x-axis.

**At low noise levels **(up to *σ*_*noise *_= 0.01), the difference between the prediction error levels of the true (*ODE*_*T*_) and the simplified (*ODE*_*S*_) kinetic models was very high, around two orders of magnitude (Figure 4). At these simulations, SPCA always performed better than *ODE*_*S *_and worse than *ODE*_*T*_, in the cross validations. At that level, forecast analysis resulted in very similar performance with very high sensitivity and specificity.

**Figure 5:**
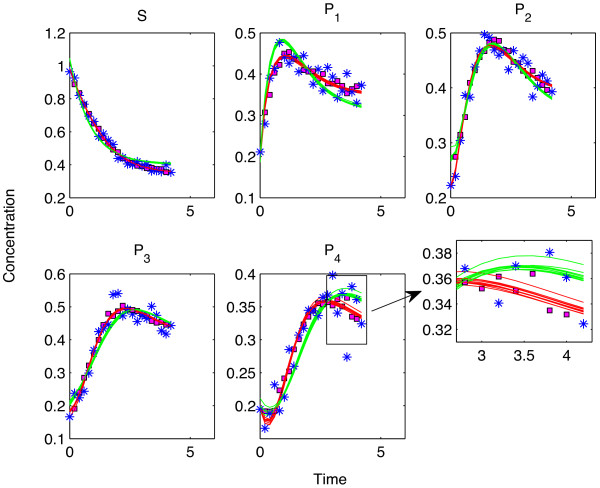
**Predictions by different models at medium noise level - Cross validation. **The blue stars denote the data points whereas the magenta squares show the SPCA predictions when the corresponding data points were excluded as test set elements. The red and the green lines show the reconstructed time profiles of the metabolites by using the true and the simplified models for different test sets, respectively. The magnifying window in the lower right hand side of the figure shows the differences of the reconstructed time profiles for different test sets. There, the deviation between the lines of the same color (obtained by using different test sets but the same model description each time) can be seen in great detail.

**At medium noise level **(*σ*_*noise *_= 0.025), the difference between prediction error levels of *ODE*_*T *_and *ODE*_*S *_became smaller due to noise interference. At that point, the reconstructed metabolite profile by *ODE*_*S *_(green line in Figure 5) pointed to a reasonable model for the data (blue stars) from a qualitative point of view. However, our quantitative analysis showed that *ODE*_*S *_predictions were worse than SPCA in the cross validations. This showed that SPCA predictions could be used to invalidate *ODE*_*S *_with very high sensitivity. Decision for not invalidating *ODE*_*T *_in most of the cases showed the specificity of the method. The number of noise realizations at which SPCA cross validation invalidated *ODE*_*T *_or *ODE*_*S *_can be seen in Table 1 for each noise level.

Up to this noise level, we determined the optimal value of the *λ* parameter as 0.005 by cross validation for all different realizations of the data. Cross validation gave also the optimal number of principal components as 4 in all of the cases covering more than 99% of the variance in the data. We estimated the optimal values of the parameters to be the same in different noise realizations due to the low amount of noise in the data. However, starting with this noise level, we had to determine the values of the SPCA parameters differently for each noise realization. This clearly showed that the datasets in 100 different noise realizations had different characteristics due to the increasing difference in the realization of the added noise. The difference in the parameters were more apparent for the forecast analysis than for the cross validation.

**Figure 6:**
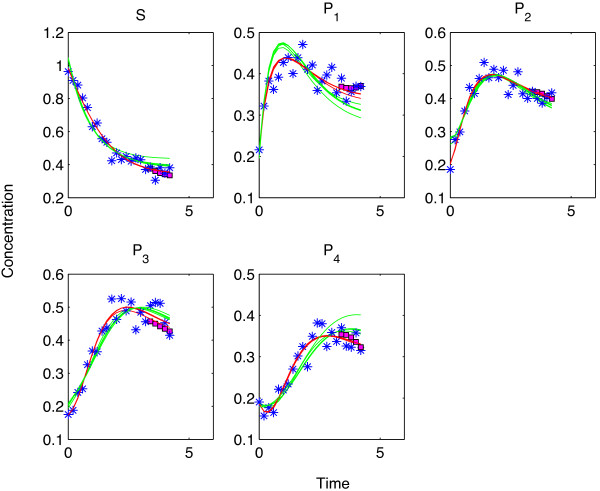
**Predictions by different models at medium noise level - Forecast analysis. **The color coding for this figure follows the one in Figure 5. In the forecast analysis approach, a window of the data which consisted of a significant number of consecutive time points were left out as test sets for each metabolite. The figure shows the SPCA predictions with magenta squares which are better than the *ODE*_*S *_and worse than the *ODE*_*T*_.

At this noise level, invalidation by forecasting started to drag behind the cross validations. Apparently, noise interfered more when consecutive time points in the end of the time profiles were removed from the training data. This held true for both the SPCA and the kinetic modeling. Due to worsening predictions of SPCA, *ODE*_*S *_could not be invalidated in 14% of the noise realizations (see Table 2). However, the predictions by the *ODE*_*T *_got also worse, resulting in an incorrect invalidation decision in 17% of the realizations. Predictions of an example simulation at this noise level can be seen in Figure 6.

**Figure 7:**
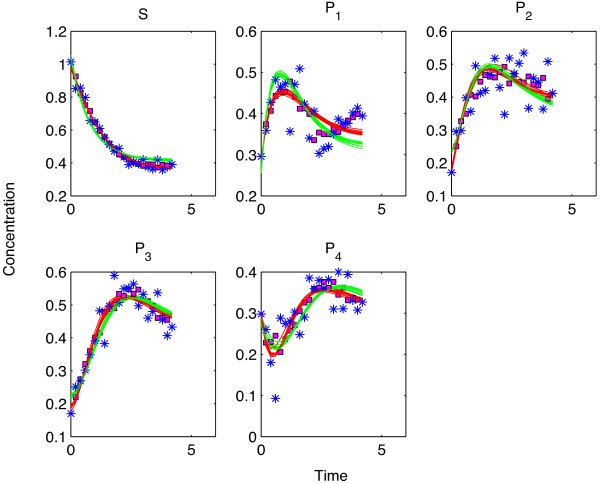
**Predictions by different models at very high noise level. **The color coding for this figure follows the one in Figure 5. At this noise level, the data seems very deteriorated by noise especially for metabolites with lower concentration ranges since the added noise is homogeneous.

**At high noise levels** (*σ*_*noise *_= 0.05), *ODE*_*T *_started to lose its predictive power compared to SPCA in 14% of the realizations (see Table 1). This could have stemmed from inefficient estimation of the model parameters because of possible local minima in the optimization. In order to check that, optimization was repeated in those problematic cases with multiple starting points. This revealed that the problem was not due to sub-optimal parameters but due to the fact that data was too deteriorated to be explained well even by *ODE*_*T *_(Figure 7). However, still in 75% of the realizations, SPCA predictions invalidated *ODE*_*S *_successfully. At this noise level, inference of the optimal SPCA parameters in the cross validations started to be affected by the noise as well. The value of the smoothing parameter *λ* and the number of PCs determined by cross validation using other test set patterns were not always optimal. That is why we adopted a grid search approach for this noise level in which we varied the parameter *λ* in a small range around the value determined by cross validation. As long as we could find better predictions by SPCA than the model in question, we could conclude that we could invalidate that model. Here, we have to emphasize that during the grid search in the small neighborhood of the estimated *λ*, SPCA predictions changed very little. This showed that prediction error from SPCA was very stable. As we use it as a threshold for invalidation of models, proving to be robust against small changes in the parameters is very important.The overall results of our simulations study with the toy model suggest that SPCA predictions within a traditional stratified cross validation framework perform very well as a threshold measure which can be used to invalidate too simple models. It meets the essential criteria of being totally unsupervised and providing a good description of the data. Even at very high levels of noise (Figure 7), it can serve as an invalidating measure. SPCA predictions within a forecasting framework also serve well for the invalidation purpose. However, it performs worse in high noise levels. On the other hand, we think that for many kinetic modelers, forecasting seems more intuitive and biologically meaningful. Therefore, it is of high importance to include it in our study.

#### ***Noise level affects the plausibility of model simplifying approximations:***

As a small demonstration of a specific research question for which our approach can be used, we investigated the plausibility of model simplifying approximations in kinetic modeling.

We used a moderate value (0.33) for the first Michaelis constant (*Km*_1_) while generating the data. Its value was well within the range of the substrate concentration ([*S*]∈[0.2,1]). If it was much higher than the substrate concentration, the substrate concentration term in the denominator of the first rate equation (see Equation 1) could have been neglected. Therefore, the model simplification from *ODE*_*T *_to *ODE*_*S *_could have been performed with very low information loss. This approximation is widely employed in many model fitting studies to justify the simplification of Michaelis-Menten Kinetics to linear kinetics which helps to decrease the number of parameters in the model. However, the ranges of the parameter values in which this approximation will be plausible are never clear.

By using our SPCA-based invalidation approach, we could investigate how the invalidation decisions changed for the simplified model with respect to the value of the Michaelis constant. This helped us to assess the plausibility of the approximation based on the degree of support by the available data. We could also observe how that assessment became difficult by increasing noise in the data. For this purpose, we used three different *Km*_1 _values in data generation. We performed the simulations with noise levels between *σ*_*noise *_= 0.01 and *σ*_*noise *_= 0.04.

We could see the expected relationship between the value of the Michaelis constant and the plausibility of the model simplifying approximation by using our approach. When the Michaelis constant was 0.33, well within the range of the substrate concentration, the simplifying approximation was never supported by the data until high amount of noise in the data (See Table 3). However, when its value was increased nearly 9-fold, well above the substrate concentration range, in all of the realizations, the data supported the simplifying approximation.

**Table 3:** The number of cases where the model simplification was acceptable

***Km***_***1***_	***σ***_***noise ***_ ***= 0.01***	***σ***_***noise ***_ ***= 0.02***	***σ***_***noise ***_ ***= 0.04***
0.33	0	0	10
1.4	44	70	82
3	100	97	94

The numbers show in how many of the 100 different noise realizations, invalidation decision could not be made for the simplified model, *ODE*_*S*_. Lack of invalidation decision showed the validity of the model simplification. The table shows that at different Michealis constant (*Km*_1_) values there is different level of support for the validity of the linear kinetics assumption.

The change in the accuracy of the plausibility assessment proved to be an even more important observation. Table 3 shows that under low levels of noise, when the Michaelis constant was only slightly above the range of the substrate concentration at 1.4, in some 40 of the realizations, *ODE*_*S *_was not invalidated. This means that the simplification was supported in nearly half of the realizations. The number of realizations at which *ODE*_*S *_could not be invalidated could increase to 82 when the measurements were more erroneous at *σ *_*noise *_= 0.04 (Mean Relative Noise ≈ 8%). This clearly shows that noise is an important factor that interferes with the plausibility of model simplification. At low noise levels, it is easier to pull out the correct kinetic mechanism from the rest of the simpler candidates. When higher noise is existent in data, detection of poorer predictions by simpler mechanisms become more difficult by the noise. Models that are in fact too simple to explain the mechanistic behaviour can be wrongly regarded as plausible candidates when the measurement accuracy is low in the experiments.

### Eicosanoid production model

**Figure 8:**
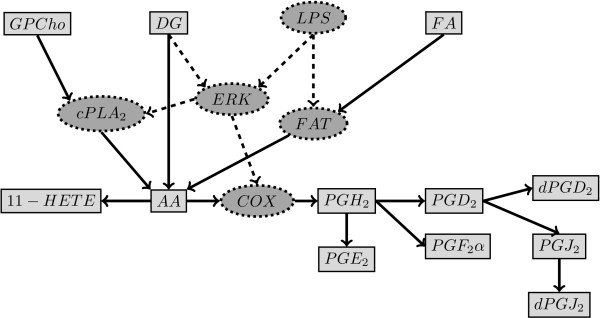
**Topology of the signaling and metabolic pathway of eicosanoid production in human.** The known pathway topology was simplified by Gupta *et al. *[2] based on the availability of metabolite concentration data in their experiments. The rectangles show the metabolites and the solid arrows indicate the metabolic transformations involved. The ellipses with dashed borders show the enzymes catalyzing the metabolic transformations between two metabolites that are neighboring it in the graph. The dashed lines denote the effect of enzymes and molecules on the activity of enzymes.

Data belonging to the biological system under study were time series concentration data (0,0.5,1,2,4,8,12,24 hours) of 8 metabolites (Arachidonic Acid, 11-HETE, PGE2, PGF2a, PGD2, PGJ2, dPGD2, dPGJ2) from 3 different experiments with 3 technical replicates (9 replicates in total) in response to treatment of human macrophage cells with KDO_2_-lipidA (an LPS analog) [2]. The model describing the system included 22 first order reaction rate parameters. The topology of the pathway is as shown in Figure 8.

We used the mean of all replicates in the calculations. However, replicates in data allowed us to estimate the noise level and we calculated the mean relative noise (MRN) in the data as 8%. That level of noise in the data corresponded to the medium to high noise level that we have covered in our simulations study. Based on the results we achieved in our simulations study, we could expect high sensitivity and specificity of our approach in that noise range.

A weighted objective function was needed in kinetic modeling to overcome the risk of it being dominated by the metabolites with higher concentrations. The weight matrix **W** we used included the reciprocal of the maximum concentration of the corresponding metabolite in all the time points. Equation 8 shows the entries of this weight matrix **W** for the training set elements. For the test elements, entries were 0 as in the case of the calculations for simulated data.

(8) $$W_{\text{ij}}=\frac{1}{\text{max}\left(X_{j}\right)}$$

In SPCA, we preprocessed the data in accordance with the kinetic modeling approach. Therefore, we first scaled every concentration value in the data matrix by the maximum concentration of the corresponding metabolite in all the time points and carried out SPCA on that scaled data matrix. It is highly recommended to scale the data prior to any type of PCA application if the order of magnitude of the data values change substantially between columns, since that will allow a more fair distribution of the loadings of the variables in the most important principal components. Then, the smoothing parameter applies more equally for every metabolite and we can achieve better smoothing of all the time profiles.We used an 8-fold diagonal cross validation scheme with 5 repetitions. The first test set involved consecutive time points from consecutive metabolites as was shown in Figure 2. The other 4 test sets involved time points with increasing intervals from different metabolites. By this approach we could achieve very diverse test sets and all data points except the first and last time points of each metabolite were included in a test set five times. We also weighted the resulting residuals by the maximum concentration before summing up to the final value and averaged by the number of repetitions.

**Figure 9:**
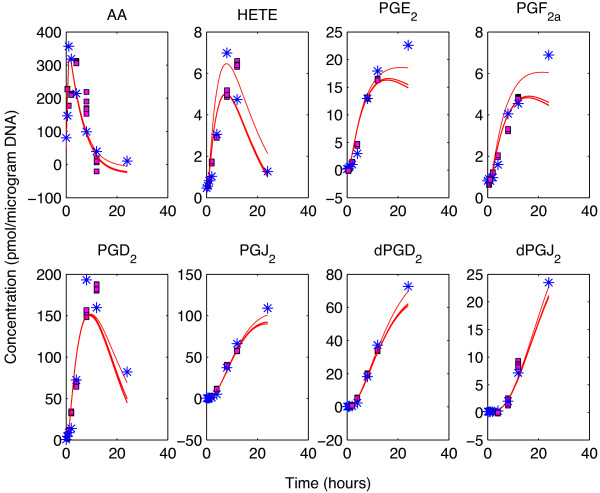
**Predictions on eicosanoid production pathway. **Solid red lines show the metabolite profiles constructed for the 8 metabolites by the kinetic model in question when different test sets were used. The blue stars show the mean of all 9 replicates of data at each time point whereas the magenta squares denote the predictions by SPCA for each data point when they were excluded from calculations as test set elements. There exist 5 SPCA predictions for each interior time point because they were included 5 times in different test sets.

The optimal *λ* and the number of principal components needed were estimated by using a 12 fold stratified cross validation scheme with 10 repetitions. We have found the optimal number of PCs to be 3 and the *λ* value between 5 and 25. Following a grid search between those lambda values, we achieved the final prediction residuals in SPCA as 6% of the sum of squares of the weighted data matrix, higher than the prediction residuals in kinetic modeling which was only 3%. These predictions can be seen in Figure 9. This showed that the model proposed for the eicosanoid production pathway could not be invalidated by using the available data. Despite its simplicity in enzymatic reaction kinetics, it proved to be competent in explaining the data.

### HOG signaling model in yeast

**Figure 10:**
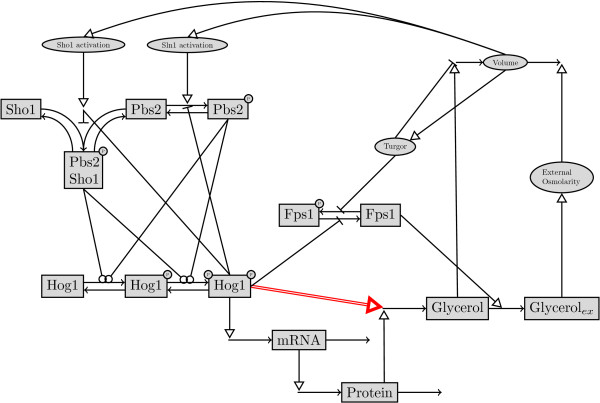
**Topology of the HOG signaling pathway in yeast. **This pathway topology was proposed as the best approximating model topology in [26]. We used this model as our true model *ODE *_*T *_for data generation in our simulations on HOG signaling pathway. The black lines with small arrow tips depict the transition between different species in the model like production, degradation or complex formation. The black lines with circle tips depict the phosphorylation process by kinases. The lines with open triangle tips show activating regulatory interactions where as lines with blunt ends show deactivating regulatory interactions. The red colored double arrow denotes the post translational regulation of glycerol production by the active phosphorylated Hog1 protein. We removed this regulatory interaction in our simplified model *ODE*_*S*_.

High osmolarity glycerol signaling pathway in yeast is a well studied system since it is regarded as a model system for studying the principles of signal transduction in eukaryotic cells. The structure of the phosphorylation cascade starting from two redundant osmosensors (Sho1p and Sln1p) and leading to the transcriptional regulation of glycerol production for osmotic balance is generally agreed upon. However there are still competing hypotheses on especially the transient feedback relations involved in the cascade. These include but are not limited to the post-translational regulation of glycerol production, Fps1p phosphorylation and Sho1p phosphorylation by the Hog1p. Schaber and coworkers carried out a comprehensive study where they compared 192 different models [26]. Here, we used their best approximating model with the accession number of MODEL1209110001 in Biomodels Database [5]. The model consisted of 15 species and 20 free parameters. 10 different variations of mass-action kinetics with either inhibitors or activators were used for the reaction kinetics in the model. Volume was also included in the model as a variable whose value changes in time. The interactions in the model can be seen in Figure 10.

#### ***Synthetic data***

We used the model depicted in Figure 10 to generate data by using the optimal parameter values determined in [26]. Synthetic data consisted of the time profiles of 4 different species measured following two different osmotic shocks at 0.4 and 0.5 M. NaCl in wild type cells. The species were the phosphorylated Hog1p, glycerol, Hog1 dependent protein (mainly Gpd1p) and the associated mRNA. We set the number of measurement points to 43 which spans the dynamic part of the profiles between the shock and the steady state at around one hour later. Following the generation of model values, we added heterogeneous noise on the data. Noise was drawn from a standard normal distribution with two different values of standard deviation and multiplied by the concentration value of the species at that time point. The standard deviation was 0.01 and 0.2 in the low and high noise levels, respectively. We carried out kinetic modeling with the true model, *ODE*_*T *_that we used to generate the data and a simplified model *ODE*_*S *_which lacked the post-translational regulation of glycerol production by the phosphorylated Hog1p (see Figure 10). During both kinetic modeling and SPCA we used a weighting matrix which normalizes the difference between the data and the model predictions, by the mean of the concentration values of the species during all the time points. Weighting serves the purposes we explained in the previous section.

**Figure 11:**
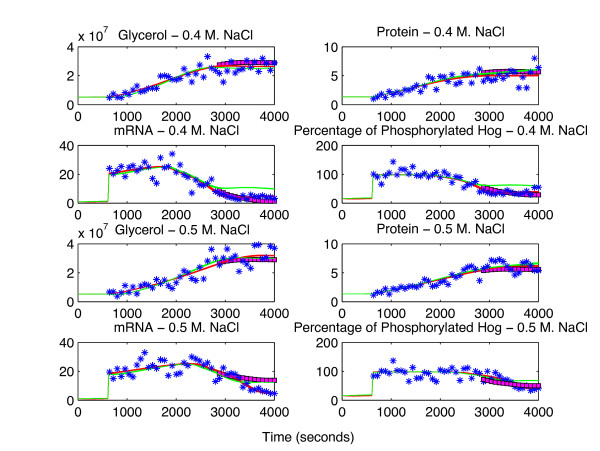
**Predictions using synthetic data on HOG signaling pathway.** In this figure, blue stars denote the synthetic data whereas magenta squares denote the SPCA predictions when the associated data points were left out as test set points. The red and the green solid lines show the time profiles predicted by the *ODE*_*T *_and the *ODE*_*S*_, respectively. The upper 4 subplots belong to the 0.4 M. NaCl shock experiment and the lower 4 subplots belong to the 0.5 M. NaCl shock experiment. The glycerol, Hog1 dependent protein (mainly Gpd1p) and mRNA amounts (in *μ*moles) are in absolute scale whereas we used the normalized phosphorylated Hog1p values. The Hog1p values were normalized to their maximum value measured in the corresponding experiment.

In this section, we employed the forecast analysis approach. In each run, we left out approximately 30% of the last time points of each species as the test set. For the determination of the optimal SPCA parameters, we followed a grid search approach and found that 2 principal components are enough with a mild smoothing penalty with *λ *= 1. In Table 4 we report the number of invalidation decisions made for the two models and Figure 11 show the kinetic model and SPCA predictions on this dataset. Our results in this section confirmed once more that SPCA can predict well even when approximately one third of the data for a single species is left out. This can be seen especially in the upper 4 plots in Figure 11 where predictions not only on the steady part but also on the dynamic part of the profile are good. Even at this very high noise level (see Figure 11), SPCA predictions in forecast analysis can serve as an invalidating measure since *ODE*_*S *_could be invalidated in all the noise realizations.

**Table 4:** All the invalidation decisions made for HOG pathway models

***σ***_***noise***_	***ODE***_***S***_	***ODE***_***T***_
0.001	100	0
0.02	100	16

The numbers show in how many of the 100 different noise realizations an invalidation decision was made for the models being questioned by using forecast analysis. *ODE*_*S *_and *ODE*_*T *_denote the simplified model and the true model, respectively.

#### ***Real data***

We used a part of the experimental data from [26] and [27] to question the best HOG signaling model reported in [26]. The real data included 4 different species. The first species was the phosphorylated Hog1p whose concentration values were normalized by its maximum concentration value in wild type cells at the same osmotic shock experiment. It was measured for the Sho1 and Sln1 deletion mutants at 6 different levels of osmotic shock. The other species were glycerol, protein and the associated mRNA measured in wild type cell following 0.5 M. NaCl treatment. Those species’ concentrations were also normalized by their corresponding maximum concentration throughout their time profiles. We used only the dynamic part of the time profiles which start after the osmotic shock. Some of the interior time points were missing in the original data so we interpolated between the existing data points to achieve a full data matrix of 13 time points and 15 columns. We needed a full data matrix because calculating the prediction residuals for the comparison of the two approaches is a very essential step in our analysis and for this purpose, we need to know the real values of the concentration values at the data points that we leave out as test sets. Therefore we imputed the missing values prior to SPCA & ODE modeling by interpolation. In total, more than half of the time points were calculated by interpolation for the Hog1 dependent proteins (mainly Gpd1p) and the glycerol. We questioned two different models as in the case of the synthetic data. The simplified model lacked the post-translational modification of glycerol production by the Hog1p.

**Figure 12:**
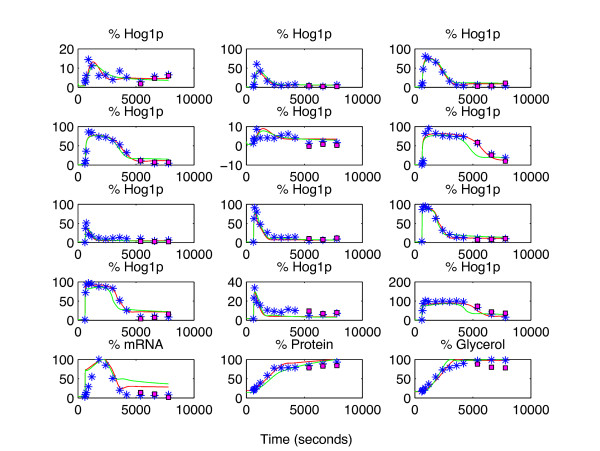
**Predictions using real data on HOG signaling pathway. **In this figure, blue stars denote the synthetic data whereas magenta squares denote the SPCA predictions when the associated data points were left out as test set points. The red and the green solid lines show the time profiles predicted by the full and the simplified models respectively. The upper 6 subplots belong to the phosphorylated Hog1p in Sln1 deletion mutant following 0.1, 0.2, 0.4, 0.6, 0.07 and 0.8 M. NaCl shock, respectively. The next 6 subplots belong to the same species in Sho1 deletion mutant after the same osmotic shocks. The last 3 subplots show the normalized mRNA, protein and glycerol concentration values.

We used forecast analysis in which we left out the last 3 time points from each column of the data matrix in each run. SPCA on this data matrix with 9 PC’s and *λ *= 8.10^6^ resulted in a very good representation of the dataset. Forecasting prediction error obtained from SPCA equals 0.6% of the sum of squares of the whole data matrix. This value was below the residuals obtained by the kinetic modeling using the full model and the simplified model, being 0.9% and 1.5% of the sum of squares of the whole data. Those predictions can be seen in Figure 12.

The results showed us that the model in question did not prove to be sufficient to explain the real data from [26] and [27] that we have used in our study. However, here we used only some part of the data that was available. Furthermore we had to impute many missing values prior to our calculations as mentioned above in this section. The reason for this is that we preferred to use the minimum amount of data that would suffice for the parameterization of the ODE model. Therefore the results we highlight here should be regarded as a more realistic demonstration of our approach rather than arriving at strict biological conclusions.

## Conclusions

We introduced the use of two resampling methods, namely cross validation and forecast analysis for the analysis of kinetic systems biology models. Cross validation and forecast analysis allowed us to use a part of the available time series metabolite concentration data to infer the proposed model’s kinetic parameters and the remaining part of the same dataset to assess the predictive power of the model. This way, we have showed that resampling strategies eliminated the need for additional datasets for the assessment of predictive capabilities of models. We used those two approaches within a Smooth Principal Components Analysis (SPCA)-based comparative approach for the invalidation of models.

Our approach depends on the assumption that correct kinetic model descriptions can predict the test data better than unsupervised data analysis methods which do not make use of any biochemical knowledge. Therefore, deficiency of a kinetic model in prediction compared to prediction by unsupervised data analysis methods tells us that the model cannot describe the data sufficiently well. A solid measure of this level of ‘sufficiency’ is needed by the biochemical modeling community because most of the time, we aim at the simplest model which is still competent in explaining the data as was also given as a guideline in [12]. On the other hand, it is very important to emphasize that this kind of comparison to unsupervised methods is only needed for the assessment of kinetic models’ validity. We do not intend to underestimate the role of kinetic modeling by showing that there can be cases where unsupervised data analysis methods are superior to some kinetic models. Every kinetic model in systems biology is valuable and deserves attention just because they aim at providing mechanistic explanations which the unsupervised data analysis methods in statistics lack. That independence from kinetic model structure is also exactly the reason why we used the predictive power of unsupervised data analysis methods as a reference point in this study. We used Smooth Principal Components Analysis for this purpose. SPCA offers better predictive capabilities than normal PCA since it can make use of also the underlying time profile and hence is more suitable for time series data. SPCA is also very robust against small changes in the smoothing parameter *λ*, proving to be a stable reference point.

With our simulations study using synthetic data generated by a toy model, we showed that until high amount of experimental noise in the data, cross validation SPCA prediction error can work as a threshold to invalidate a too simple kinetic model with high specificity and sensitivity. It is however important to note that for an accurate comparison of predictive power, the inferred parameters of the kinetic model have to be optimal. Although proven to be not an easy task, there are many methods proposed in the literature to overcome the local minima problems encountered [38-40] during parameter inference.

Forecast analysis requires higher penalties for smoothing of the scores in SPCA and noise is more influential. Predictions by SPCA forecasting and kinetic modeling are more dependent on the noise realization in the data compared to cross validation with interior time points. Therefore, we need to be more aware of the estimated noise level in the data if we want to use SPCA forecasting prediction error as an invalidation measure.

Our SPCA-based invalidation approach can also be employed iteratively for model reduction. Analyses of model families derived from a master model has proved to be a popular approach in biochemical modeling [11,12,26,41]. In this approach, a master model is allowed to be manipulated in certain directions, either by changing the interactions and the species involved or changing the kinetic laws of the model. By this way, a very high number of models with very different number of parameters are created and analyzed. Here, selection of the best model within the large family of models is a critical task. Our invalidation approach can be very useful in that stage. The most complex models within the model family can be questioned first for their validity. Later, they can be subject to step-wise simplification by removal of interactions or simplification of reaction kinetics. At a certain stage, the models would be invalidated by our approach meaning that they fail to explain the data sufficiently well. This would mean that the models are in their simplest acceptable form one step before the invalidation decision. However, at that step there would still be a number of models with different characteristics which could not be invalidated. Therefore, the problem of model invalidation turns to a problem of model selection between a number of models with similar complexities. Therefore, at that point, we can make use of model selection criteria like AIC or BIC complementary to our invalidation approach for the ultimate selection of the best model.

## Competing interests

The authors declare that they have no competing interests.

## Authors’ contributions

DH, HCJH and JAW conceived and designed the study. DH wrote the software, performed the calculations and wrote the manuscript. HCJH and AKS supervised the study and helped to draft the manuscript. All the authors read and approved the manuscript.
